# To what extent could performance-based schemes help increase the effectiveness of prevention of mother-to-child transmission of HIV (PMTCT) programs in resource-limited settings? a summary of the published evidence

**DOI:** 10.1186/1471-2458-10-702

**Published:** 2010-11-16

**Authors:** Hapsatou Touré, Martine Audibert, François Dabis

**Affiliations:** 1 INSERM U897, Institut de Santé Publique d'Épidémiologie et de Développement (ISPED), Université Victor Segalen, 146 Rue Léo Saignat, 33000, Bordeaux, France; 2 CNRS, Centre d'Etudes et de Recherches sur le Développement International (CERDI), Université d'Auvergne, 65 Boulevard François Mitterrand, 63000, Clermont-Ferrand, France

## Abstract

**Background:**

In resource-limited settings, HIV/AIDS remains a serious threat to the social and physical well-being of women of childbearing age, pregnant women, mothers and infants.

**Discussion:**

In sub-Saharan African countries with high prevalence rates, pediatric HIV/AIDS acquired through mother-to-child transmission (MTCT) can in largely be prevented by using well-established biomedical interventions. Logistical and socio-cultural barriers continue, however, to undermine the successful prevention of MTCT (PMTCT). In this paper, we review reports on maternal, neonatal and child health, as well as HIV care and treatment services that look at program incentives.

**Summary:**

These studies suggest that comprehensive PMTCT strategies aiming to maximize health-worker motivation in developing countries must involve a mix of both financial and non-financial incentives. The establishment of robust ethical and regulatory standards in public-sector HIV care centers could reduce barriers to PMTCT service provision in sub-Saharan Africa and help them in achieving universal PMTCT targets.

## Background

The risk of mother-to-child transmission (MTCT) of HIV has declined from 35% to less than 2% thanks to the development of biomedical interventions that can be provided to women and/or children during pregnancy, labor, delivery and breastfeeding [1,2]. Vertical HIV transmission-the main route of pediatric HIV infection-has thus virtually disappeared in high-income settings, where integrated and comprehensive strategic services for prevention of MTCT (PMTCT) have been successful and are now the standard of care [3]. Conversely, vertical HIV transmission remains an important public health issue in the developing world because the scale-up of PMTCT services so far has been slow and highly uneven [4-7]. Too few pregnant women in low- and middle-income countries are aware of their HIV status. In 2009, only 26% of women attending antenatal care received an HIV test, and 53% of those who were found to be HIV-infected received an antiretroviral prophylaxis for PMTCT, which rarely contained the most powerful combinations of antiretroviral drugs (ARV) [8]. An estimated 430,000 children were newly infected with HIV in 2008-over 90% of them in sub-Saharan Africa [9]. Without treatment, an estimated half of these infected children will die before their second birthday [10].

There is no doubt that PMTCT is a key strategy for the achievement of the Millennium Development Goals 4, 5 and 6. PMTCT-sometimes referred to as the four-pronged strategy-is more than the simple administration of ARVs. Indeed, a full set of key interventions is recommended to be implemented as an integral component of essential maternal, neonatal and child health (MNCH) services [11]. The first and second prongs emphasize the important role of primary prevention of HIV among women of reproductive age and the need to prevent unintended pregnancies among HIV-infected women. The third prong of the strategy targets pregnant women already infected and recommends that HIV testing is integrated in MNCH units where ARVs are provided to prevent infection being passed on to their babies and also to improve the woman's own health; it requires also that adequate counseling is provided regarding the best feeding option for the baby. The fourth and last prong calls for better integration of HIV care, treatment and support for women found to be HIV infected and for their families (Table 1).

**Table 1:** Four-prong strategy for a comprehensive approach of the prevention of mother-to-child transmission (PMTCT) of HIV

1st prong	Prevention of HIV infection among young persons and pregnant women
2^nd ^prong	Prevention of unintended pregnancies in HIV-infected women
3^rd ^prong	Prevention of HIV transmission from HIV-infected women to their infants
4^th ^prong	Provision of treatment, care, and support to HIV-infected women and their families

The evidence from developed countries suggests that integrated health delivery systems may be a cost-effective way to meet the health-care needs of a population. The sexual and reproductive health specialists have been calling for integration with HIV services for many years. An initial focus on the integration of HIV prevention and testing services into family planning, antenatal and postnatal care in the mid-1990 s was followed by a drive to integrate PMTCT within MNCH services. Besides, the United Nations Millennium Development Goals incorporate a target of universal access to sexual and reproductive health within the goal of improving MNCH services-but fighting HIV often remains a separate project with malaria and tuberculosis [12]. A review of the Global Fund database for elements and indicators of sexual and reproductive health in all approved HIV-related proposals-submitted by 134 countries, from rounds 1 to 7-retrieved that only 20% to 30% of them included sexual health counseling, gender-based violence, and the linking of voluntary counseling and testing for HIV with sexual and reproductive health services [13].

Integrating PMTCT care into MNCH services and linking mother and child HIV status are necessary for HIV-infected mothers and HIV-exposed children to receive appropriate follow-up and treatment. Nonetheless, competing demands for resources within the health-care system require health-care providers to ensure the most effective and efficient use of resources. As we move towards eliminating pediatric HIV infection through PMTCT in resource-limited countries, it is imperative to improve the quality of the existing health-care workforce. In this paper, we review the literature on incentives-especially pay-for-performance schemes-and human resource management (HRM) in HIV care and treatment programs, particularly those that offer PMTCT. We seek to determine the impact of incentives and HRM on the effectiveness and quality of care provided by health-care workers in resource-limited countries.

## Discussion

### Human resources issues

#### Health workforce shortage

In fact, low- and middle-income countries not only have the highest rates of HIV worldwide, but also face a severe shortage of health-care workers. Sub-Saharan Africa is home to 11% of the global population and nearly two-thirds of people living with HIV/AIDS, but only 3% of the world's health-care workers [14]. Staff shortages in resource-limited settings are a major obstacle to the scale-up of HIV care and treatment, including PMTCT. In 2006, the World Health Organization (WHO) was one of the first international bodies to devote an entire report to the global health-care workforce crisis, thus renewing interest in this topic [14]. The shortage of trained health care providers in resource-limited settings has existed for decades, but in recent years the number of workers has been inadequate at almost all levels of the health system. One reason is that the number of people completing health-related training has not kept up with the increasing needs [15]. It is therefore critical to use the available health-care personnel in a cost-effective manner.

An estimated 45 countries worldwide, of which an overwhelming majority are in sub-Saharan Africa, will not meet the WHO target of skilled health-care attendants delivering 80% of the live births by 2015 [16]. The added workload brought on by the HIV epidemic has increased the strain on fragile health systems and already over-stretched health-workers [17,18]. In Tanzania, the average staff workload at MNCH clinics ranges from 37.8% at facilities that do not provide PMTCT to 50.5% at clinics in which trained health-care workers provide PMTCT services [19]. This suggests that health professionals may be less motivated to undertake PMTCT-related activities. A performance-based system for providing PMTCT services may therefore improve MNCH and long-term HIV care and treatment.

#### Motivation: definition and association with performance

According to economic theories, individuals only choose to work if the overall benefits of their labor outweigh the associated opportunity costs. Benefits may include financial and non-financial rewards [20,21]. Professional motivation is defined as an individual's willingness to work towards the goals of an organization. In order for health-care workers to be effective, they must be motivated and willing to implement the reforms proposed by health policy makers [22]. The problem of under-performance and de-motivation of health-workers has been often explained in terms of an asymmetric principal-agent relationship where agents (health-workers) lack incentives to perform and principals (employers and patients) lack sanction and accountability mechanisms [23]. HRM and support systems can increase motivation by improving working conditions and involving staff in management decisions. Reviews of health-care worker motivation in resource-limited settings have shown that a wide range of factors on both the individual and group level, as well as in various social and cultural contexts, are likely to influence their expectations and effectiveness when providing care. However, these studies have rarely assessed HIV care or PMTCT specifically [24-27].

**Figure 1:**
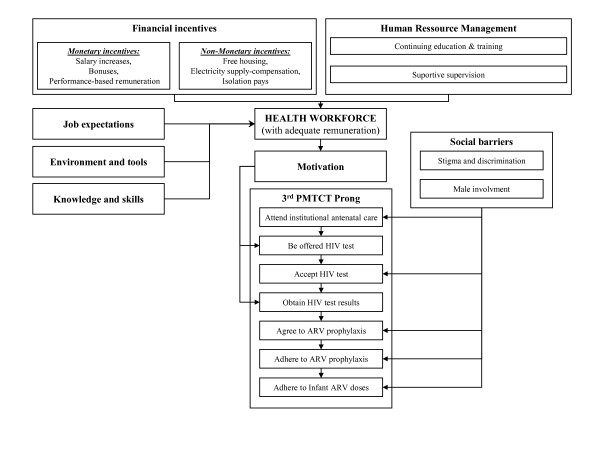
**Potential impact of incentives on prevention of mother-to-child HIV transmission (PMTCT) services**.

The WHO has put forth four criteria for evaluating performance of health-care workers: availability, competence, responsiveness and productivity [14]. Several international development organizations have also adapted a "Performance Improvement Model" for use in low- and middle-income settings. This model outlines five key factors that influence performance outcomes: job expectations; performance feedback; environment and tools; motivation and incentives; and knowledge and skills. One group used this model in Armenia to assess the factors affecting performance among MNCH health-care providers [28]. They found that among people providing antenatal and postpartum care, performance was associated with practical knowledge and the ability to use everyday tools, recognition for good work, and performance feedback. These findings suggest that a set of incentives could help in introducing and scaling-up effective PMTCT programs (Figure 1).

#### Where do we stand on the concept of incentives?

First, without adequate remuneration, health-care workers will not perform their tasks effectively, nor will they commit to their jobs on the long term, regardless of their responsibilities and initial level of commitment [29,30]. It may therefore be possible to create explicit financial incentives, for instance by increasing salaries or providing bonuses to those working in underserved communities or rural regions [31].

Although wage concerns are important, they are not the only problem for already over-stretched health-care workers. Other important factors include professional fulfillment and opportunities for career advancement. Non-financial incentives may include compensation payments that improve living conditions, such as free housing, transportation or electricity, professional support and supervision, streamlined management, opportunities for continuing education and training, relocations, or promotions [32,33] (Figure 1). However, it is important to note that non-financial incentives weigh as much as the financial ones and both need to be linked together.

### Summary of experiences in resource-limited settings

#### Strategies to improve provision of PMTCT services

One potential approach to improve PMTCT coverage is opt-out, provider-initiated HIV testing and counseling (PITC) [34]. HIV screening practices are currently shifting from voluntary, patient-initiated testing to routine, provider-initiated testing that includes group pre-test counseling, a rapid HIV test, individual post-test counseling, and encouragement of community and spousal involvement [35,36]. PITC has thus become the standard practice in many resource-limited countries: in 2008, of the 53 countries with generalized HIV epidemics that reported information on PITC, half reported that its introduction had encouraged health-care workers to propose testing and counseling to everyone, regardless of presenting symptoms and type of facility [37].

Another method for improving the provision of PMTCT services may be the formal reorganization of tasks among health-care workers. International bodies such as the WHO and the United States President's Emergency Plan For AIDS Relief (PEPFAR) advocate alternative models of care delivery that rely more on nurses and lay health-workers than physicians [38]. Reports from rural and primary health-care centers in Rwanda, Lesotho and elsewhere demonstrate that nurses can prescribe antiretroviral treatment (ART) safely and effectively to HIV-infected adults and children as long as they receive adequate training and support [39-43]. A cost-minimization model has also shown that task-shifting during the follow-up of patients on ART can substantially reduce costs as well as the need for physicians. Replacing the standard physician-intensive follow-up with nurse-intensive follow-up, for instance, could save 90 full-time-equivalent physicians, or 4.1% of the national physician workforce in Uganda [44]. Innovative staffing strategies that use off-duty workers, trained traditional birth attendants and HIV-infected female community health-care workers to provide MNCH and PMTCT services could help alleviate the shortage of human resources in many MNCH centers [45-48].

Despite the growing use of PITC and alternative models of care delivery, translating PMTCT research findings into practice remains difficult in resource-limited settings [49]. A carefully thought-out package of incentives must be proposed to health professionals, to ensure that they not only continue working in the field, but also agree to relocate to rural areas if necessary. Indonesia has successfully attracted physicians to peripheral islands and retained them there, using a financial incentive package. Once they complete their public service requirement, health-workers who agree to work in very remote areas receive higher salaries -isolation pay-as well as a guaranteed career in the civil service, which is highly valued because it allows for complementary private practice and free access to specialist training [50].

Patients can also receive incentives to present to care. In rural Nepal, midwives received financial incentives to outreach pregnant women in neighboring villages [51]. Pregnant women were also reimbursed for the cost of transportation to the clinic if they gave birth with the help of skilled health-care attendants. The authors of this study acknowledged that close supervision and strong relations between the target community and the health-care workers probably contributed to the success of this program. Furthermore, the sustainability of such incentives over time is unknown and results likely depend on context.

#### Salary

Public health-care worker salaries are set according to local budgets, which rely on the national budget and international funding agencies, rather than worker productivity. As a result, public sector health-care workers are often paid too little compared to the local cost of living and generally develop individual strategies to increase their incomes [29].

Many studies have shown that financial incentives are rarely the only factor that motivates staff and improves performance. In fact, health-care workers may agree to work for less pay if their job is perceived to be attractive enough [31,32]. Few studies, however, have evaluated the quality of supervision and continuing education, which could both help improve the quality of health services over time.

#### Importance of HRM and local context

Financial incentives are particularly important when salaries are very low. Health-care workers are at risk, however, of considering them as bonuses rather than incentives. Even when salaries are reasonable, financial incentives alone are often not enough. A study conducted in public and private facilities in rural Benin and Kenya evaluated the impact of non-monetary incentives. It showed that health-workers were strongly guided by their professional conscience and ethos [52]. Many health-workers were unmotivated and frustrated, because of the lack of means and supplies, as well as inadequate or inappropriately used HRM tools such as supervision, recognition, training, career planning, and leadership, preventing them from pursuing their vocation and reaching their full potential. A study on the association between motivation and performance among health-care workers in Mali showed that after salary, responsibility, training and recognition were the main motivators [53]. Health-care workers declared that they gained professional satisfaction from receiving recognition for their work, having responsibilities, and performing the work itself. Factors that led to discontent were related to poor working conditions such as inadequate equipment, no job description, and subjective performance evaluation. Interviewees added that their motivation would likely increase if they had more responsibilities and recognition for their work. Access to and funding for education and promotion is also important for motivating and retaining workers [26,33,54,55]. These published reports confirm our hypothesis that non-monetary incentives and HRM tools play an important role in motivating health professionals.

Financial incentives have not been shown to improve patient health when health systems and their personnel are not equipped to handle the necessary quantitative and qualitative changes. For instance, the role of traditional birth attendants (TBAs) in MNCH centers and uptake of PMTCT services is unclear, with some studies reporting an increase and others reporting no change in uptake after implementation of TBA-directed incentives [46,56]. Furthermore, a system of incentives and allowances that differs from those at non-HIV health centers could lead the most qualified personnel to "defect" to HIV care programs, thus reducing the quality of health services in other sectors [57].

#### Performance-based experiences in MNCH and HIV/AIDS

In Haiti, a United States Agency for International Development (USAID)-funded bilateral health project used pay-for-performance to increase the quantity and quality of health services. The project was monitored and evaluated, and showed that paying for results could also work in a resource-limited country. This pilot program started in 1999 in three facilities. It gradually shifted from reimbursing the health centers for documented expenditures- *pay-for-input *- to paying them in part based on their attainment of specific performance targets- *pay-for-performance*. Over the six-year phasing-in of this payment-for-performance scheme, the health centers showed remarkable improvements in a range of performance indicators, including the percentage of pregnant women who attend prenatal visits at least three times and the percentage of deliveries conducted by trained attendants [58]. Performance for health facilities in performance-based payment improved substantially, moving from provision of at least three prenatal care visits to 31% of pregnant women in the first contract period to an average of 60% in 2005. A slight increase in the proportion of assisted trained deliveries from 78.5% to 80% was concomitantly observed.

A pilot program in Rwanda demonstrated that performance-based financing for HIV services such as voluntary counseling and testing (VCT) is also feasible and sustainable in sub-Saharan Africa [59]. The program emerged from an agreement between the Rwandan Ministry of Health, USAID, and PEPFAR and aimed to improve access to and quality of HIV care throughout the country [59]. In the first nine months, HIV VCT at targeted health centers increased by 155%. It is important to note, however, that independent funding agencies oversaw the district health system at a decentralized level and ensured the separation of purchases, service delivery, and regulation by local health authorities from contract negotiation to fund disbursement. Local community groups used patient surveys to monitor the performance of health facilities as well as consumer satisfaction. These new structures provided an opportunity to redistribute roles within the Rwandan health system and make it more transparent and efficient. The performance-based financing initiative showed an increase in health services use, financial accessibility, and health-care worker motivation. By incorporating the private sector into the health system, it also strengthened public-private partnerships in Rwanda. More to the point, in the province of Cyangugu, a performance-based financing initiative aiming to create checks and balances at the district level, showed good results in terms of motivation of health staff, financial accessibility as well as use of services. In fact, not only out-of-pocket health expenditure decreased by 62% from US$ 9.05 to US$ 3.45 but more outstandingly the proportion of women delivering in a health facility concomitantly increased from 25% to 60%-offering a good opportunity of linkage with PMTCT services [60].

The Rwandan experience may provide lessons for other countries, as it shows that the combination of output-based payment and greater autonomy is a feasible and effective strategy for improving the performance of public health services at a local level [61]. Nonetheless, the Rwandan experiment has been described by the targeted health-care workers as having put additional stress on an already overstretched health-care system. Some staff members also complained of the ethical issues associated with choosing between activities seen as necessary (e.g. intensive care) and those required to attain performance targets (e.g. filling out forms) [62]. Moreover, this might disrupt the whole system by making health professionals turn away from perceived under-financed infectious diseases to HIV-related activities, for instance.

#### What about performance-based schemes in PMTCT?

Few studies have explored performance-based financing for PMTCT. In 2008, the Elizabeth Glaser Pediatric AIDS Foundation (EGPAF) initiated a pilot performance-based financing scheme in collaboration with private and faith-based health facilities in Côte d'Ivoire, the country with the highest HIV prevalence in West Africa. Côte d'Ivoire did not have a national framework for performance-based financing at that time. EGPAF recently reported the first results of this initiative, but its sustainability over time remains to be assessed [63]. In brief, the program team designed 22 indicators to evaluate performance-based financing of PMTCT and HIV care and treatment. When they compared data from the year before initiating the program to data from the year after its inception, they found that performance-based financing was associated with improved quantity and quality of HIV prevention, care and treatment, as well as a 100% increase in health-care worker effort. To work, performance-based schemes should be built upon reliable monitoring and evaluation systems that routinely collect process data allowing evaluation of the strategy implemented. The effectiveness of performance-based financing of PMTCT, its sustainability, and its impact on the efficacy of PMTCT have never been practically evaluated on a global scale.

### Concluding remarks

Over-paying professionals to encourage them to commit to their job may pose ethical problems. In low- and middle-income settings, however, public health sector wages are rarely associated with education level or cost of living. Few studies in resource-limited settings have evaluated the effects of financial and non-financial incentives on performance in the public health sector, especially in the field of HIV and PMTCT. The implementation and evaluation of large-scale operations are critical at a time when international and bilateral agencies are pushing to attain ambitious PMTCT targets that remain distant in many countries [64]. Resource availability and worker competence are essential for meeting these goals, but they will not be sufficient to ensure that health-care providers work as efficiently as required. Increasing worker motivation requires consideration of many factors that cross disciplinary boundaries-economics, psychology, sociology and human resource management. Financial incentives may be important determinants of worker motivation for PMTCT, but they cannot resolve all worker motivation problems alone. Human resource management-one indicator of health-care workers' commitment to their jobs-should be considered in the design and implementation of PMTCT performance-based initiatives, which are urgently needed to eliminate the pediatric HIV/AIDS epidemic.

### Summary

PMTCT in sub-Saharan Africa has encountered several programmatic barriers that impeded the full translation of biomedical research findings at the population level. One of the main challenges to PMTCT coverage is the shortage of health-care workers. Performance-based financing schemes involving robust human management systems may help increase motivation among selected health-care workers. The effectiveness of performance-based financing of PMTCT on a global scale depends on context, but its long-term sustainability remains unknown.

## Competing interests

The authors declare that they have no competing interests.

## Authors' contributions

MA and HT designed the study. HT drafted the initial article and finalized it after review. MA and FD commented extensively. All authors were involved in the revision of the paper and approved the final version.

## Pre-publication history

The pre-publication history for this paper can be accessed here:

http://www.biomedcentral.com/1471-2458/10/702/prepub
